# Trait profiling and genotype selection in oilseed rape using genotype by trait and genotype by yield*trait approaches

**DOI:** 10.1002/fsn3.3290

**Published:** 2023-02-28

**Authors:** Behnam Bakhshi, Hassan Amiri Oghan, Valiollah Rameeh, Hossein Zeinalzadeh Tabrizi, Abdolhossein Askari, Abolfazl Faraji, Gholamreza Ghodrati, Hamid Reza Fanaei, Amir Khosro Danaei, Narjes Khatoon Kazerani, Kamal Payghamzadeh, Davood Kiani, Hossein Sadeghi, Farnaz Shariati, Alireza Dalili, Mohammad Ali Aghajani Nasab Afrouzi

**Affiliations:** ^1^ Horticulture Crops Research Department, Sistan Agricultural and Natural Resources Research and Education Center AREEO Zabol Iran; ^2^ Seed and Plant Improvement Institute, Agricultural Research Education and Extension Organization (AREEO) Karaj Iran; ^3^ Horticulture Crops Research Department, Mazanderan Agricultural and Natural Resources Research and Education Center AREEO Sari Iran; ^4^ Department of Horticulture and Agronomy, Faculty of Agriculture Kyrgyz‐Turkish Manas University Bishkek Kyrgyzstan; ^5^ Horticulture Crops Research Department, Hormozgan Agricultural and Natural Resources Research and Education Center AREEO Bandar Abbas Iran; ^6^ Horticulture Crops Research Department, Golestan Agricultural and Natural Resources Research and Education Center AREEO Gorgan Iran; ^7^ Horticulture Crops Research Department, Safi‐Abad Agricultural and Natural Resources Research and Education Center AREEO Dezful Iran; ^8^ Horticulture Crops Research Department, Khozestan Agricultural and Natural Resources Research and Education Center AREEO Behbahan Iran; ^9^ Horticulture Crops Research Department, Boshehr Agricultural and Natural Resources Research and Education Center AREEO Borazjan Iran; ^10^ Seed and Plant Certification and Registration Institute Agricultural Research, Education and Extension Organization (AREEO) Karaj Iran; ^11^ Plant Protection Research Department, Mazanderan Agricultural and Natural Resources Research and Education Center AREEO Sari Iran; ^12^ Plant Protection Research Department, Golestan Agricultural and Natural Resources Research and Education Center AREEO Gorgan Iran

**Keywords:** GT biplot, GYT biplot, high‐yielding, multi environmental trial

## Abstract

Selection and breeding for high‐yielding in oilseed rape have always been one of the leading objectives for oilseed rape breeders. This process becomes more complicated when all quantitative traits are considered in selection in addition to grain yield. In the present study, 18 oilseed rape genotypes along with 2 check cultivars (RGS003 and Dalgan) were evaluated across 16 environments (a combination of 2 years and eight locations) in the tropical climate regions of Iran during 2018–2019 and 2019–2020 cropping seasons. The experiments were conducted in a format of randomized complete block design (RCBD) with three replications. The obtained multienvironmental trial data were utilized to conduct multivariate analysis, genotype by trait (GT) biplot, and genotype by yield*trait (GYT) biplot (Breeding, Genetics and Genomics, 1:2019). The GT and GYT biplot accounted for 55.5% and 93.6% of the total variation in the first two main components. Based on multivariate analysis and GT biplot, pod numbers in plant (PNP) and plant height (PH) were chosen as two key traits in spring oilseed rape genotypes for indirect selection due to high variation, strong positive correlation with grain yield (GY), and their high representatively and discriminability in genotype selection. The mean × stability GT biplot represented G10 (SRL‐96‐17) as the superior genotype. Based on the mean × stability GYT biplot, eight above‐average genotypes were identified that took high scores in stability, high‐yielding, and all evaluated quantitative traits at the same time. Based on the superiority index of GYT data, G10 (SRL‐96‐17) and G5 (SRL‐96‐11) indicated the best yield–trait combinations profile and ranked above check cultivars and then selected as superior genotypes. Similarly, cluster analysis using the WARD method also separated eight superior genotypes. Based on the result of the present study, GT ad GYT methodologies are recommended for trait profiling and genotype selection in oilseed rape breeding projects, respectively.

## Graphical abstract

Pod number in plant and plant height were detected as the most essential selection criteria for grain yield enhancement in oilseed rape. GT ad GYT methodologies are recommended for trait profiling and genotype selection in oilseed rape breeding projects, respectively. The methodology of cluster analysis based on standardized GYT data led us to separate traits based on their direct and indirect effects in addition to genotype selection.

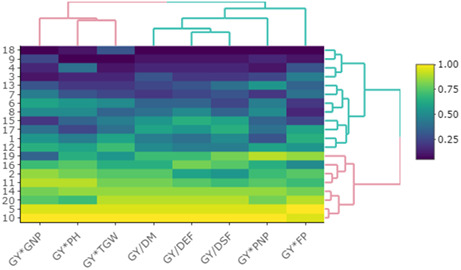

## 1 INTRODUCTION

Oilseed rape (*Brassica napus* L.) is the second worldwide major supplier of edible oil, after soybeans (Liersch et al., 2020). The oilseed rape grain production reached almost 71 million tons during the 2018–2019 cropping season (Liersch et al., 2020). In Iran, however, oilseed rape is the first major oilseed cultivated crop (Amiri Oghan et al., 2016). Based on reported data in FAOSTAT, 289996 t of oilseed rape was harvested in Iran from an area of approximately 140,000 ha during the 2019–2020 cropping season, with an average yield of about 2074 kg/ha (FAO, 2018).

Most of the previous studies of oilseed rape genotypes selection have been focused on yield (Escobar et al., 2011; Miah et al., 2015; Rahnejat & Farshadfar, 2015; Shojaei et al., 2011; Zali et al., 2016). Grain yield is a complex trait and the main purpose of plant breeders is to identify the foundation of relationships between grain yield and other traits to increase grain production (Zulfiqar et al., 2021). Achieving high yields is one of the most important goals in plant breeding programs. However, there are three key challenges in the process of genotype evaluation that limit the success of plant breeders: first genotype by environment interaction (GE), second the reverse or unfavorable relationship among key traits, and third the high complexity of the key traits like yield (Kendal et al., 2019; Sofi et al., 2022; Yan & Frégeau‐Reid, 2018).

Different models are developed to overcome this challenge. The first is independent culling which discards genotypes fail to meet the minimum required value for a trait, regardless of how well the genotype is for other traits. The second is index selection which ranks genotypes based on index values of a linear combination of the target traits (Sofi et al., 2022; Yan & Frégeau‐Reid, 2018). The major difficulty associated with these strategies is their high subjectivity. The weight and truncation points can potentially vary from researcher to researcher and from time to time for the same researcher, even for the same dataset (Yan & Frégeau‐Reid, 2018).

Regarding GGE‐biplot analysis, new methods are developed like a genotype‐by‐trait (GT) biplot which visualizes the genotype relationship with traits in a biplot to represent the strengths and weaknesses of the genotypes (Yan & Rajcan, 2002). This methodology has been widely used by many researchers to find the traits relationship in different plants (Dehghani et al., 2008; Gouveia et al., 2022; Karahan & Akgun, 2020; Santana et al., 2021; Sofi et al., 2022; Tsenov et al., 2021).

Despite the advantages of GT biplot in identifying the interrelationships among the traits of genotypes and trait profiles, this methodology could not prepare enough results for the breeders about the selection or elimination of genotypes. To wipe out these deficiencies and increase the efficiency of genotype selection, the GYT biplot methodology was developed (Yan & Frégeau‐Reid, 2018). This methodology afforded a novel comprehensive and effective approach to evaluate the genotypes based on multiple traits through a graphical ranking of genotypes. The feature of this strategy is combining yield with various target traits which made it possible to represent the strengths and weaknesses of the genotypes at the same time. Based on the GYT biplot, yield is considered the constant trait in the determination of the efficiency of a genotype by itself, while other traits are valuable to producers only when they are combined with sufficiently good yield levels (Yan & Frégeau‐Reid, 2018).

In the present study, 18 spring oilseed rape genotypes along with the two check cultivars were studied based on GT and GYT approaches. This study aimed to define the interrelationship among the traits, the association among genotypes and traits, and the ranking of genotypes based on multiple traits.

## 2 MATERIALS AND METHODS

### 2.1 Plant materials and multienvironment trials

A total of 20 open‐pollinated spring oilseed rape genotypes (Table 1) were evaluated for their quantitative traits including grain yield (GY), days to start flowering (DSF), days to end flowering (DEF), days to maturity (DM), flowering period (FP), plant height (PH), pod numbers in plant (PNP), grain numbers in pod (GNP), one thousand grain weight (TGW). The experiment has been conducted across 16 environments (combination of years and locations) in the tropical climate of Iran including north and south tropical regions (Gorgan, Sari, Moghan, Behbahan, Borazjan, Dezfoul, Zabol, and Hajiabad) during 2018–2019 and 2019–2020 cropping seasons in a format of randomized complete block design (RCBD) with three replications. More description of these environments is presented in Table 2. Each plot consisted of four rows five‐meter long with a spacing of 30 cm between rows and 5 cm within rows. The spacing of 60 cm was considered between the plots. Seeds of all genotypes were sown with an experimental sowing machine. The amount of seed consumption was 6 kg/ha which was sown according to the instructions on the suitable dates in each region.

**TABLE 1:** Code, name, and origin of the tested oilseed rape genotypes.

No	Code	Name	Origin
1	G1	SRL‐96‐6	Iran
2	G2	SRL‐96‐7	Iran
3	G3	SRL‐96‐8	Iran
4	G4	SRL‐96‐10	Iran
5	G5	SRL‐96‐11	Iran
6	G6	SRL‐96‐12	Iran
7	G7	SRL‐96‐13	Iran
8	G8	SRL‐96‐15	Iran
9	G9	SRL‐96‐16	Iran
10	G10	SRL‐96‐17	Iran
11	G11	SRL‐96‐18	Iran
12	G12	SRL‐96‐19	Iran
13	G13	SRL‐96‐20	Iran
14	G14	SRL‐96‐21	Iran
15	G15	SRL‐96‐22	Iran
16	G16	SRL‐96‐23	Iran
17	G17	SRL‐96‐24	Iran
18	G18	SRL‐96‐25	Iran
19	G19	RGS003	Iran
20	G20	Dalgan	Iran

**TABLE 2:** Agro‐climate description of the tested environments.

Code	Location	Cropping season	Longitude (E)	Latitude (N)	Altitude (m)	Average temperature (°C)	Accumulated rainfall (mm)	Mean yield (kg/ha)
E1	Sari	2018–2019	53° 13′	36° 46′	15	16.2	435	2624
E2	Sari	2019–2020	53° 13′	36° 46′	15	14.9	511	3000
E3	Gorgan	2018–2019	54° 24′	36° 53′	5	15.6	643.1	2418
E4	Gorgan	2019–2020	54° 24′	36°53′	5	15.34	465.1	2052
E5	Moghan	2018–2019	47° 94′	39° 36′	74	12.57	188.3	3610
E6	Moghan	2019–2020	47° 94′	39° 36′	71	12.36	159.5	3050
E7	Zabol	2018–2019	61° 40′	30° 54′	492	17.4	70.8	3429
E8	Zabol	2019–2020	61° 40′	30° 54′	492	16.5	112.3	2959
E9	Borazjan	2018–2019	51° 15′	29° 12′	106	19.5	342.5	2855
E10	Borazjan	2019–2020	51° 15′	29° 12′	106	21.6	390.7	2501
E11	Dezful	2018–2019	48° 32′	32° 22′	82	25.64	215.1	1824
E12	Dezful	2019–2020	48° 32′	32° 22′	82	24.83	615	1871
E13	Behbahan	2018–2019	50° 14′	30° 36′	320	18.03	546	2514
E14	Behbahan	2019–2020	50° 14′	30° 36′	320	18.28	311.9	2523
E15	Hajiabad	2018–2019	55° 52′	28° 18′	920	16.62	242	2897
E16	Hajiabad	2019–2020	55° 52′	28° 18′	920	17.71	198	2963

### 2.2 Statistical analysis

In this study, 18 new promising oilseed rape spring genotypes along with 2 check spring cultivars were evaluated. Multivariate analysis including Pearson correlation, Ward cluster, and path analysis was conducted in this study to find the relationship between the assessed genotypes traits.

#### 2.2.1 Genotype by trait (GT) biplot methodology

The data from 16 environments (a combination of 2 years and eight locations) were utilized to prepare the genotype by trait (GT) biplot (Yan & Tinker, 2006) and genotype by yield*trait (GYT) biplot (Yan & Frégeau‐Reid, 2018). The Genotype‐by‐trait (GT) table was created by the mean value of 16 environments' data for each genotype trait. Genotype‐by‐trait (GT) biplot methodology visualizes traits and genotype relationships in a biplot to represent the strengths and weaknesses of traits and genotypes (Yan & Rajcan, 2002). Based on the GT biplot, acute, obtuse, and right angles of traits vectors represent positive, negative and zero (no) correlation, respectively. Furthermore, a relatively short vector of the GT biplot indicates the low variation of traits across genotypes and vice versa (Yan & Rajcan, 2002).

#### 2.2.2 Genotype by yield*trait (GYT) biplot methodology

Using the GT table, the genotype by yield*trait (GYT) biplot (Yan & Frégeau‐Reid, 2018) was constructed in this study. To provide the GYT table, each trait of the GT table is multiplied or divided by grain yield based on the breeding objectives as follows: for traits which their high values are desirable including flowering period (FP), plant height (PH), pod numbers in plant (PNP), grain numbers in pod (GNP), one thousand grain weight (TGW), each of them multiplied with the grain yield (e.g., GY*PNP), while for traits which their high values are undesirable including days to start flowering (DSF), days to end flowering (DEF), days to maturity (DM), each of them divided with the grain yield (e.g., GY/DSF). Thus, a larger value in the GYT table is always more desirable.

The GYT data were standardized for each yield–trait combination as represented in the following formula and then integrated to calculate the superiority index (SI) for each genotype (Yan & Frégeau‐Reid, 2018). The high value of SI indicated its superiority (Yan & Frégeau‐Reid, 2018).

Standardized yield*trait value = $\frac{\text{Genotype yield}\_\text{trait value}-\text{Yield}\_\text{trait mean of}\;\mathrm{all}\;\text{genotypes}\;}{\text{Yield}\_\text{trait Standard deviation of}\;\mathrm{all}\;\text{genotypes}}$

### 2.3 Utilized software

The GT and GYT data were analyzed using Excel and GGEbiplot software (Yan & Kang, 2002). Correlation, cluster, and path analysis were conducted using Corrplot (Wei et al., 2017), Heatmaply (Galili et al., 2018), and Lavaan (Oberski, 2014) R statistical software packages, respectively.

## 3 RESULTS

### 3.1 Traits profiling based on Pearson correlation and path analysis

**FIGURE 1:**
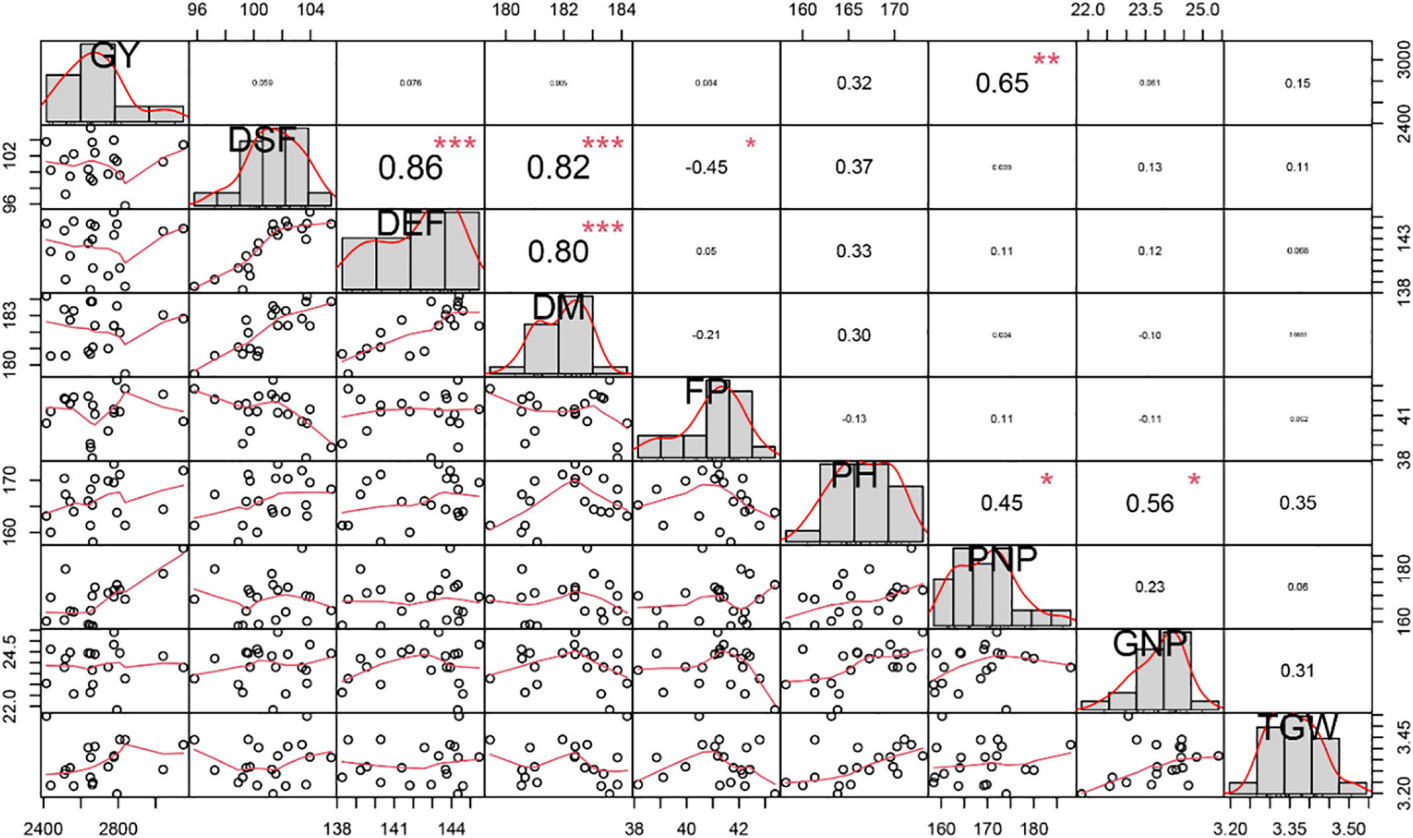
The Pearson correlation among the evaluated traits of 20 oilseed rape genotypes. DEF, days to end flowering; DM, days to maturity; DSF, days to start flowering; FP, flowering period; GNP, grain numbers in pod; GY, grain yield; PH, plant height; PNP, pod numbers in plant; TGW, one thousand grain weight.

**FIGURE 2:**
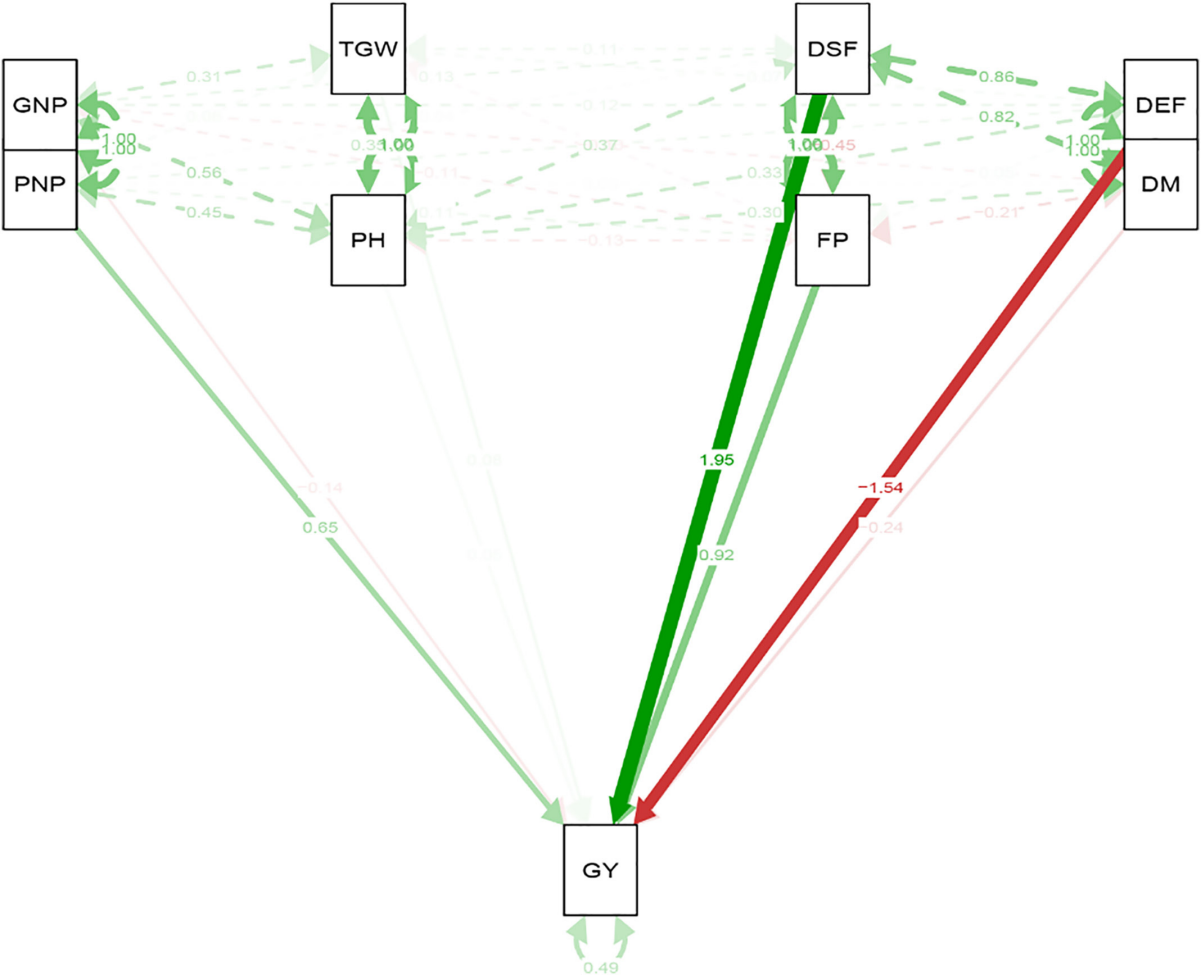
Path analysis for evaluated traits of 20 oilseed rape genotypes. DEF, days to end flowering; DM, days to maturity; DSF, days to start flowering; FP, flowering period; GNP, grain numbers in pod; GY, grain yield; PH, plant height; PNP, pod numbers in plant; TGW, one thousand grain weight.

The mean of traits (genotype by trait) data across 16 environments (combination of 2 years and eight locations) for 20 oilseed rape genotypes are shown in Table 3. The highest variation belonged to pod numbers in plant (PNP), grain numbers in pod (GNP), and grain yield (GY), respectively. The Pearson correlations among all evaluated traits were conducted and presented in Figure 1. Based on the Pearson correlation, strong positive correlations were observed among all phenological traits including days to start flowering (DSF), days to end flowering (DEF), and days to maturity (DM). The flowering period (FP) was negatively correlated with days to start flowering (DSF). Based on this, flowering period (FP) would be increased in the early flowering genotypes. Plant height (PH) was also positively correlated with pod numbers in plant (PNP) and grain numbers in pod (GNP). Grain yield (GY) also showed a strong positive correlation with pod numbers in plant (PNP). Additionally, based on path analysis, days to start flowering (DSF), days to end flowering (DEF), flowering period (FP), and pod numbers in plant (PNP) indicated a direct effect on grain yield (GY), while other quantitative traits like plant height (PH), one thousand grain weight (TGW), and grain numbers in pod (GNP) represented an indirect effect on grain yield (GY) (Figure 2).

**TABLE 3:** The mean of traits (genotype by trait) data across 16 environments (combination of 2 years and eight locations) for 20 oilseed rape genotypes.

Genotype	GY (kg/ha^−1^)	DSF (date)	DEF (date)	DM (date)	FP (day)	PH (cm)	PNP (count)	GNP (count)	TGW (g)
G1	2640	100.3	142.6	180.8	42.3	166.0	159.0	24.4	3.4
G2	2777	104.0	145.5	182.4	41.4	169.6	169.5	24.8	3.4
G3	2562	102.3	144.6	183.3	42.2	164.0	163.7	22.6	3.2
G4	2512	101.5	143.8	183.4	42.1	170.4	160.6	23.8	3.3
G5	3040	101.3	143.7	183.0	42.4	164.4	178.2	24.3	3.3
G6	2653	105.5	144.4	183.9	38.9	168.3	164.3	24.4	3.4
G7	2543	99.5	141.4	182.7	41.8	165.9	164.0	24.5	3.3
G8	2662	103.7	142.9	183.9	38.1	165.3	169.8	23.7	3.2
G9	2439	100.2	141.8	180.5	41.3	160.0	169.2	24.6	3.2
G10	3148	103.4	143.9	182.8	40.6	171.9	187.9	23.8	3.4
G11	2778	101.7	143.3	182.4	41.2	173.1	172.0	25.4	3.4
G12	2675	102.4	144.2	182.4	41.1	170.3	173.1	24.4	3.4
G13	2518	97.2	139.2	180.6	42.1	167.3	180.0	24.2	3.3
G14	2808	99.6	140.3	182.0	41.3	171.1	172.2	24.4	3.4
G15	2651	99.2	138.3	180.7	39.1	161.3	158.6	22.6	3.3
G16	2746	99.7	139.6	181.0	40.0	169.9	170.9	23.8	3.3
G17	2664	98.9	140.3	181.1	41.7	158.2	158.4	23.0	3.2
G18	2416	103.8	144.4	184.2	40.5	163.1	160.2	23.1	3.5
G19	2793	101.4	144.3	183.6	43.4	163.8	174.1	21.8	3.2
G20	2837	95.8	138.6	179.5	42.8	161.3	168.5	23.3	3.4
Mean	53,863	2021.2	2847.0	3644.0	824.2	3325.1	3374.5	477.0	66.7
LSD	126.21	1.06	1.10	0.90	1.23	3.49	9.76	1.05	0.08
CV	11.69	2.63	1.92	1.23	7.44	5.23	14.43	10.95	6.12

Abbreviations: DEF, days to end flowering; DM, days to maturity; DSF, days to start flowering; FP, flowering period; GNP, grain numbers in pod; GY, grain yield; PH, plant height; PNP, pod numbers in plant; TGW, one thousand grain weight.

### 3.2 Genotype by trait (GT) biplot

**FIGURE 3:**
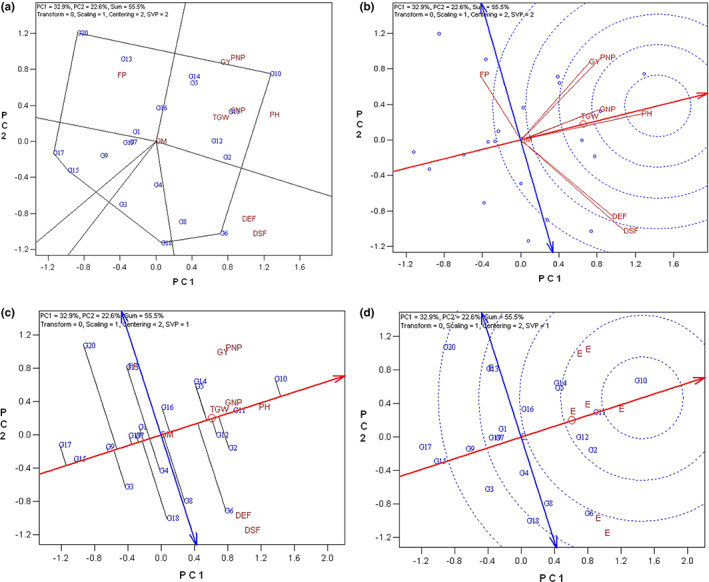
Genotype by trait (GT) biplots; (a) The which‐won‐where biplot; (b) Ranking traits biplot based on both discriminating and representativeness; (c) Mean × stability biplot; (d) Ranking genotypes biplot based on both mean and stability. DEF, days to end flowering; DM, days to maturity; DSF, days to start flowering; FP, flowering period; GNP, grain numbers in pod; GY, grain yield; PH, plant height; PNP, pod numbers in plant; TGW, one thousand grain weight.

The trait‐standardized genotype by trait (GT) data was utilized in the current study to represent the trait profiles by GT biplot. The GT biplot accounted for 55.5% of the total variation of data. The first and second principal components accounted for 32.9% and 22.6% of the variation in data, respectively (Figure 3).

#### 3.2.1 Uncovering the relationship of traits based on GT biplot

To grouping the studied traits and genotypes, the polygon view or which‐won‐where biplot of GT data is demonstrated in Figure 2a. Which‐won‐where GT biplot is created by connecting the longest vectors of the genotypes in all directions. A line from the origin of the biplot is perpendicular to each side of the polygon to divide the yield–traits combinations into different sectors. The genotypes placed at the vertex of each sector have the largest values of the included traits (Santana et al., 2021; Yan et al., 2000; Yan & Tinker, 2006). Based on this, the polygon was divided into 6 sectors. The vertex genotypes were G10 for pod numbers in plant (PNP), grain yield (GY), plant height (PH), grain numbers in pod (GNP), one thousand grain weight (TGW), and days to maturity (DM); G6 for days to end flowering (DEF) and days to start flowering (DSF); and G20 for the flowering period (FP). Genotype G10 is located at the vertex of the sector composed of most of the traits. Thereby, this genotype had high values of the included traits and could define as the superior genotype (Figure 3a).

Based on the GT biplot, the cosine of the angles between two trait vectors determines the intensity of correlation. Therefore, acute (smaller than 90°), obtuse (greater than 90°), and right (exact 90°) angles represent positive, negative, and zero (no) correlation between traits, respectively. Grain yield (GY) vector formed acute angle with pod numbers in plant (PNP), grain numbers in pod (GNP), one thousand grain weight (TGW), plant height (PH), and flowering period (FP) vectors which represent their positive correlations. Grain yield (GY) and pod numbers in plant (PNP) vectors were too close to each other which emphasizes their strong positive correlation. On the contrary, days to start flowering (DSF) and days to end flowering (DEF) vectors showed no correlation with the grain yield (GY) vector.

#### 3.2.2 Determining ideal traits to identify superior genotypes

The studied traits were ranked based on both discriminating ability and representativeness. The length of the trait vectors shows how well the trait is represented in the biplot. The greater trait vector indicates more discrimination of the trait, while the relatively short vector demonstrates an insufficient variation of the trait across genotypes (Santana et al., 2021; Yan & Frégeau‐Reid, 2018). Consequently, pod numbers in plant (PNP), plant height (PH), days to start flowering (DSF), and days to end flowering (DEF) could be considered discriminative traits due to their long vectors; however, pod numbers in plant (PNP) and plant height (PH) might be more effective in grain yield incensement due to their positive correlation with grain yield (GY). Besides, the acute angle between the trait vector and ATC (Average Tester Coordination) represents greater representativeness. Based on this, plant height (PH), which is located in the center of concentric circles, could be considered the most discriminative and representative trait to identify superior genotypes (Figure 3b).

Additionally, to identify an ideal genotype, the GT data were also visualized based on the ATC view of the biplot (Figure 3d). Based on this, the center of concentric circles defines as an ideal trait and the nearest genotypes to this point would be the superior genotypes. Eventually, genotype G10 that is the nearest to the center of concentric circles considered as the superior one.

#### 3.2.3 Genotype ranking based on the mean × stability GT biplot

The mean × stability biplot was also used in this study to compare genotypes based on GT data (Figure 3c). The small circle in the ATC view of the GT biplot indicates the average of traits. A single arrow line termed the average tester axis (ATA) points to higher mean values of all traits passing through the origin and the small circle of the biplot. A double‐arrowed line (perpendicular to the ATA) passes through the biplot origin and separated the better‐than‐average genotypes (right side of the double‐arrowed line) from those poorer than average (left side of the double‐arrowed line). Based on this, the best ranked above average (right side of the double‐arrowed line) genotypes included: G10 > G11 > G2 > G12 > G5 > 14 > G6 > G16 > G8. This finding shows that genotype G10 would be the superior genotype. Moreover, genotypes with short projections to the double‐arrowed line placed close to ATA and tend to have balanced trait profiles; on the other hand, those with obvious strengths or weaknesses in their trait profiles placed away from ATA in either direction (Santana et al., 2021; Yan et al., 2000; Yan & Tinker, 2006). Therefore, genotypes G6 and G8 were powerless among the right side ones considering their quantitative traits like grain yield (GY), pod numbers in plant (PNP), grain numbers in pod (GNP), plant height (PH), and one thousand grain weight (TGW).

### 3.3 GYT biplot

**FIGURE 4:**
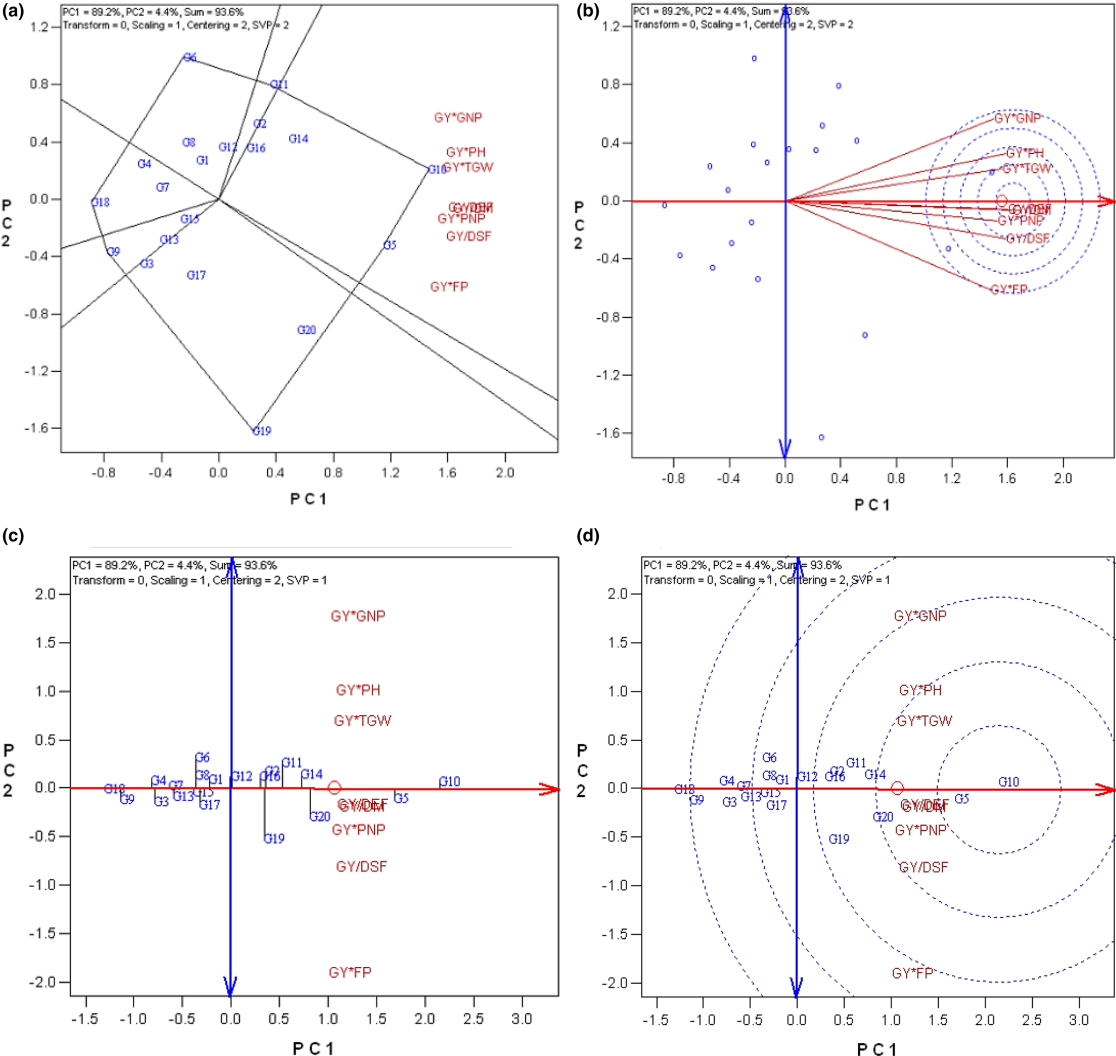
Genotype by yield*trait (GYT) biplots; (a) The which‐won‐where biplot; (b) Ranking traits biplot based on both discriminating and representativeness; (c) Mean × stability biplot; (d) Ranking genotypes biplot based on both mean and stability. DEF, days to end flowering; DM, days to maturity; DSF, days to start flowering; FP, flowering period; GNP, grain numbers in pod; GY, grain yield; PH, plant height; PNP, pod numbers in plant; TGW, one thousand grain weight.

The original GT data (Table 3) were used to create the GYT table through a combination of grain yield (GY) with quantitative traits (Table 4). To this end, traits whose larger values were desirable were multiplied by grain yield (GY), while the traits whose lower values were favorable (including earliness traits) were divided by grain yield (GY). Therefore, a larger value in the GYT table is always desirable. The data of the GYT table were applied to a graphical display named GYT biplot. Principal component analysis indicated that the first and second principal components accounted for 93.6% of the total variation in the yield–trait combinations data. The first principal component represents 89.2% of the total variation of the data and the second principal accounted for 4.4% of the total variation of the data (Figure 4).

**TABLE 4:** Genotype by yield*trait (GYT) data, its standard values and the genotypes ranking based on superiority index for evaluated oilseed rape genotypes in this study.

Genotype	GYT data								GYT standardized data								Superiority index	RANK
	GY/DSF	GY/DEF	GY/DM	GY*FP	GY*PH	GY*PNP	GY*GNP	GY*TGW	GY/DSF	GY/DEF	GY/DM	GY*FP	GY*PH	GY*PNP	GY*GNP	GY*TGW		
G1	26.3	18.5	14.6	111,646	438,269	419,737	64,537	8988	−0.19	−0.31	−0.18	0.07	−0.27	−0.73	0.06	0.01	−0.19	10
G2	26.7	19.1	15.2	115,006	470,853	470,641	68,963	9336	0.03	0.13	0.44	0.46	0.63	0.31	0.92	0.51	0.43	6
G3	25.1	17.7	14.0	108,192	420,119	419,480	57,816	8284	−0.85	−0.93	−0.79	−0.32	−0.77	−0.73	−1.25	−1.01	−0.83	17
G4	24.7	17.5	13.7	105,872	427,954	403,489	59,774	8259	−1.02	−1.12	−1.07	−0.59	−0.56	−1.06	−0.87	−1.05	−0.92	18
G5	30.0	21.2	16.6	128,952	499,894	541,857	73,900	10,053	1.78	1.72	1.81	2.06	1.44	1.77	1.88	1.54	1.75	2
G6	25.2	18.4	14.4	103,076	446,410	435,835	64,811	8914	−0.80	−0.42	−0.35	−0.91	−0.04	−0.40	0.11	−0.10	−0.36	12
G7	25.6	18.0	13.9	106,260	421,882	417,102	62,225	8428	−0.58	−0.72	−0.86	−0.54	−0.72	−0.78	−0.39	−0.80	−0.68	16
G8	25.7	18.6	14.5	101,527	439,829	451,956	62,968	8622	−0.53	−0.23	−0.30	−1.09	−0.23	−0.07	−0.25	−0.52	−0.40	13
G9	24.3	17.2	13.5	100,650	390,255	412,712	60,047	7891	−1.24	−1.33	−1.26	−1.19	−1.60	−0.87	−0.82	−1.58	−1.24	19
G10	30.4	21.9	17.2	127,835	541,051	591,723	74,881	10,759	2.01	2.28	2.41	1.93	2.58	2.80	2.07	2.56	2.33	1
G11	27.3	19.4	15.2	114,414	480,854	477,870	70,581	9354	0.35	0.35	0.44	0.39	0.91	0.46	1.24	0.53	0.59	5
G12	26.1	18.6	14.7	109,916	455,595	463,176	65,264	9122	−0.28	−0.28	−0.11	−0.12	0.21	0.16	0.20	0.20	0.00	9
G13	25.9	18.1	13.9	105,979	421,265	453,324	60,938	8320	−0.40	−0.64	−0.83	−0.58	−0.74	−0.04	−0.64	−0.96	−0.60	15
G14	28.2	20.0	15.4	115,850	480,534	483,653	68,659	9662	0.82	0.85	0.65	0.56	0.90	0.58	0.86	0.98	0.77	4
G15	26.7	19.2	14.7	103,664	427,671	420,517	60,003	8673	0.03	0.19	−0.11	−0.84	−0.56	−0.71	−0.83	−0.45	−0.41	14
G16	27.5	19.7	15.2	109,732	466,486	469,402	65,373	9114	0.47	0.58	0.39	−0.15	0.51	0.29	0.22	0.19	0.31	8
G17	26.9	19.0	14.7	111,074	421,456	422,037	61,288	8656	0.15	0.05	−0.07	0.01	−0.74	−0.68	−0.57	−0.47	−0.29	11
G18	23.3	16.7	13.1	97,789	394,112	387,019	55,692	8562	−1.79	−1.69	−1.65	−1.52	−1.49	−1.40	−1.67	−0.61	−1.48	20
G19	27.6	19.4	15.2	121,157	457,577	486,200	60,986	8932	0.48	0.33	0.43	1.17	0.27	0.63	−0.63	−0.08	0.32	7
G20	29.6	20.5	15.8	121,392	457,670	478,111	66,012	9756	1.57	1.19	1.01	1.19	0.27	0.47	0.35	1.11	0.90	3

#### 3.3.1 Determining ideal yield–trait combinations to identify superior genotypes

The which‐won‐where GYT biplot is divided into seven sectors (Figure 4a). It was observed that all yield–trait combinations were placed in the same sector with the genotype G10 at the vertex of the sector. This finding shows that genotype G10 was the best in combining grain yield (GY) with other quantitative traits.

As shown in Figure 4b, yield–trait combinations located in the center of concentric circles including GY*PNP, GY/DM, and GY/DEF represent the greater representativeness. Additionally, while there are in the middle of all yield–trait combinations, their correlation with the other yield–trait combinations is balanced. Therefore, GY*PNP, GY/DM, and GY/DEF could be defined as ideal yield–trait combinations to identify superior genotypes.

#### 3.3.2 Genotype ranking based on the mean × stability GYT biplot and the superiority index

Based on the mean × stability GYT biplot, the best ranked above average (right side of the double‐arrowed line) genotypes included: G10 > G5 > G20 > G14 > G11 > G2 > G19 > G16 (Figure 4c). Among these superior genotypes, G10 and G5 located in the center of concentric circles show the best yield–trait combinations profile (Figure 4d).

The mean of standardized yield–trait combinations values of each genotype shows its superiority index (SI). The high value of SI indicated the superiority of the genotype (Yan & Frégeau‐Reid, 2018). Based on SI values, genotypes are ranked and represented in Table 4. The ranking of SI values was similar to the results of mean × stability biplot ranking.

#### 3.3.3 Cluster analysis of GYT data for grouping the genotypes

**FIGURE 5:**
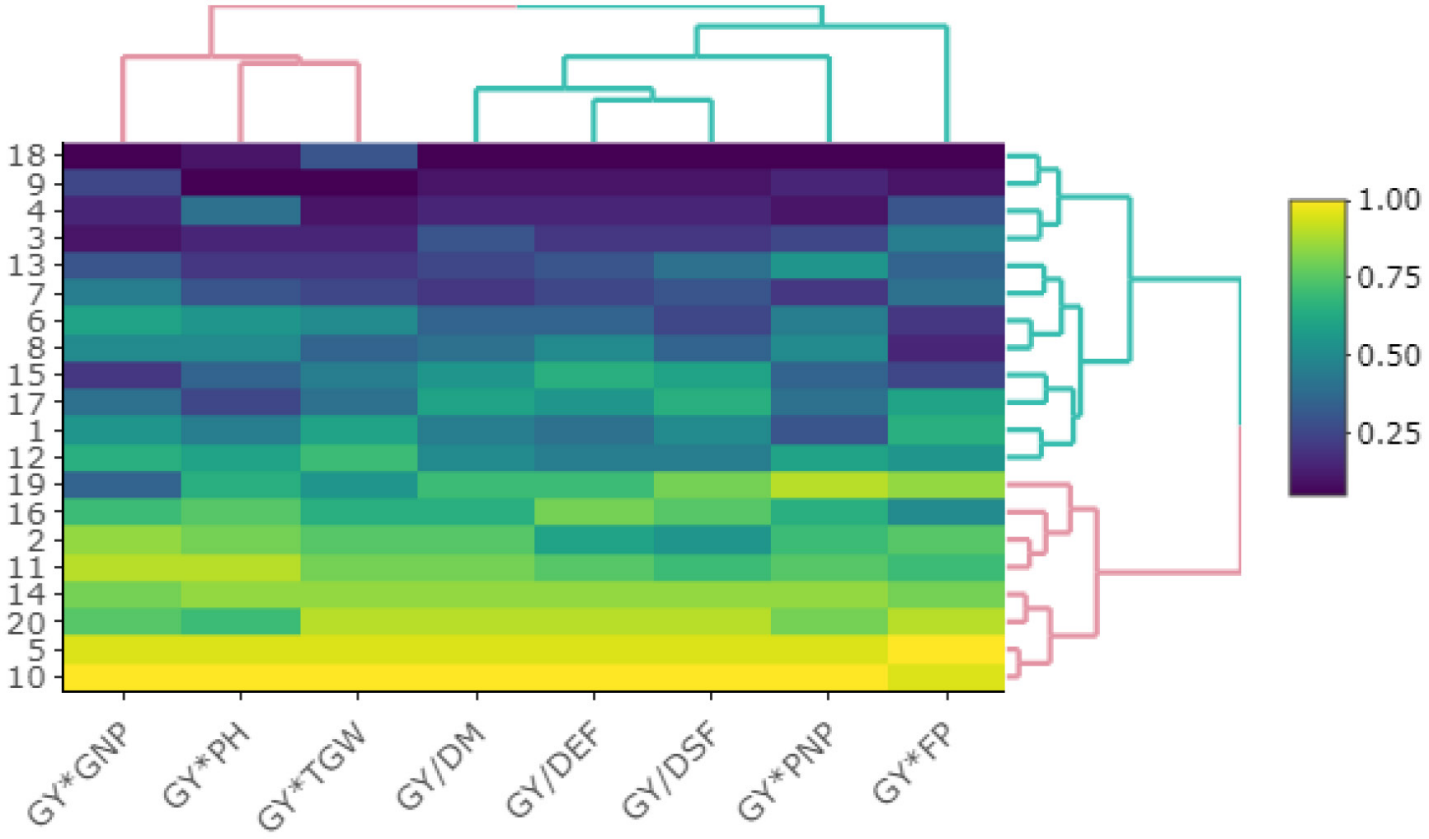
Cluster analysis of genotypes and yield–trait combinations based on standardized GYT data using the WARD method. DEF, days to end flowering; DM, days to maturity; DSF, days to start flowering; FP, flowering period; GNP, grain numbers in pod; GY, grain yield; PH, plant height; PNP, pod numbers in plant; TGW, one thousand grain weight.

Cluster analysis was conducted in this study for 20 studied oilseed rape genotypes based on standardized GYT data (Figure 5). Cluster analysis separated genotypes into two main groups. The first cluster is composed of eight genotypes with high values of yield–trait combinations; including G10, G5, G20, G14, G11, G2, G19, and G16. These eight genotypes were ranked as above‐average genotypes based on the mean × stability GYT biplot. On the contrary, the second group was composed of lower‐than‐average scored genotypes based on mean × stability GYT biplot. Therefore, cluster analysis based on standardized GYT data could be an efficient method to identify above‐average genotypes similar to mean × stability GYT biplot.

## 4 DISCUSSION

The breeding of oilseed rape, which aims at achieving higher yield, becomes a complex activity due to the negative correlation among quantitative traits. Therefore, selecting the superior genotypes considering multiple traits is one of the major challenges for oilseed rape breeders. This study aimed to identify the relationship between oilseed rape traits and ranking genotypes based on mega‐traits. To this end, a multienvironmental trial experiment was conducted in 16 environments (a combination of eight locations and 2 years) using genotype by trait (GT) and genotype by yield*trait (GYT) biplot approaches.

In this study, nine quantitative traits were evaluated. There is a wide variation among the studied genotypes, as represented by the high standard deviation values (Table 2). Pod numbers in plant (PNP), grain numbers in pod (GNP), and grain yield (GY) represented a higher variation among the studied genotypes, suggesting a wider selection range for these traits. The characteristic of CV is its no relationship with any unit of measurement (Santana et al., 2021) which made it an efficient method to comprise the studied traits.

Based on multivariate analysis, we defined pod numbers in plant (PNP) and plant height (PH) as two key traits in spring oilseed rape genotypes selection. Pod numbers in plant (PNP) were found as ideal traits due to meeting criteria including (1) strong positive correlation with grain yield (GY) based on Pearson correlation, (2) direct effect on grain yield (GY) based on path, and (3) highest variation among the evaluated traits. Plant height (PH) was also detected as the second ideal trait due to meeting criteria including (1) a strong positive correlation with pod numbers in plant (PNP) and grain numbers in pod (GNP) based on Pearson correlation and (2) indirect effect to grain yield (GY) trough yield components traits and phenological traits. Accordingly, pod numbers in plant (PNP) and plant height (PH) were detected as key traits in the genotype selection of spring oilseed rape.

GT biplot methodology has been introduced for a long time as a practical strategy for trait profiling and genotype selection in plants based on the interaction between genotypes by traits (Yan & Rajcan, 2002). However, rare studies utilized oilseed rape genotypes in this context. Dehghani et al. utilized the GT biplot for the first time using two environments (sowing date) for five winter oilseed rape genotypes (Dehghani et al., 2008). No other studies applied these approaches in oilseed rape genotypes.

GT biplot represented the relationship between traits and trait profiles of the genotypes (Yan & Rajcan, 2002). Based on the GT biplot, pod numbers in plant (PNP) and plant height (PH) were the most discriminative traits with a positive correlation to grain yield (GY). On the other hand, correlation analysis also confirmed the positive correlation of pod numbers in plant (PNP) and plant height (PH) with grain yield (GY). Instead of these traits, 1000 grain weight was previously found as the closest trait to grain yield in winter oilseed rape (Dehghani et al., 2008). However, the negative correlation of grain yield with phenological traits including days to start flowering and days to end flowering was previously confirmed in winter oilseed rape (Dehghani et al., 2008). PH was also found as the most representative trait in addition to its discriminability. Therefore, PNP and PH were also found as ideal traits for genotype selection based on the GT biplot.

Based on the GT biplot, genotype G10 was found as a superior one. This methodology ranked G2, G12, G5, G14, G6, G16, and G8 as the next superior genotypes that were better than average in their traits profile.

The negative or no relationship of grain yield with some quantitative traits like days to start flowering and days to end flowering complicated genotype selection based on traits profiling. On the other hand, the high direct impact of the phenological traits on grain yield (GY) based on Path analysis emphasized that these traits should not be neglected. Additionally, although grain yield (GY) is the major target trait, an equivalent value to other traits is allocated to it. The purpose of plant breeding is to select a genotype with desirable traits as long as they have a good yield (Yan et al., 2019). Based on this paradigm, a new approach that combined yield with the other target traits was developed named genotype by yield*trait (GYT) biplot that completes the deficiencies encountered in the GT biplot (Yan & Frégeau‐Reid, 2018). This methodology led us to rank oilseed rape genotypes based on their general advantages over yield by trait combinations. Recent studies of GT and GY*T approaches comparison reported GY*T biplot is more effective in case of multitrait and multienvironment selection as compared to a normal GT biplot (Karahan & Akgun, 2020; Sofi et al., 2022).

The GYT methodology allowed us to constitute a composition between grain yield and each trait, regarding high‐yielding genotypes as desirable ones. Thus, the desired genotypes not only were superior in their grain yield but also in terms of the other traits. Accordingly, genotypes G10, G5, G20, G14, G11, G2, G19, and G16 that were above average were identified as superior genotypes using the GYT biplot.

A comparison of the total ratio of PC1 and PC2 in total variation indicated a higher value in the GYT biplot (93.6) than GT biplot (55.5), which was consistent with a previous report on barley genotypes (Kendal, 2020). This made the GYT biplot ranking and other related outputs more reliable than the GT biplot.

Based on the results of the GYT biplot, all yield–traits combination vectors are close to each other that demonstrate their positive correlation. This is another advantage of the GYT biplot compared with the GT biplot that aligns all traits to make a strong association through combination with the grain yield (GY) (Yan & Frégeau‐Reid, 2018). Thus, genotypes could be ranked based on yield–trait combinations and superior ones could be selected with much higher accuracy considering all the traits.

The other benefit of using the GYT method is the selection of a limited number of important traits for evaluation to reduce the evaluation costs in the field (Mohammadi et al., 2020). Our results indicated a strong correlation among all yield–trait combinations. Therefore, similar results would be achievable by measuring the fewer number of these traits.

Cluster analysis was also conducted based on genotype by yield*trait data that separated the superior genotypes similar to the GYT biplot and superiority index. Accordingly, the second cluster of genotypes (vertical clustering) included series that all categorized as above average based on the GYT biplot. Additionally, the second cluster of yield*trait combination (horizontal clustering) is composed of traits with direct effects on grain yield (GY) based on path analysis. Therefore, this methodology of cluster analysis led us to separate traits based on their direct and indirect effects in addition to genotype selection.

## 5 CONCLUSION

This study aimed to determine how quantitative traits of oilseed rape genotypes are associated to increase grain yield. Another purpose of this study was to identify superior genotypes through composition grain yield with quantitative traits for the tropical regions of Iran. In this study, 20 spring oilseed rape genotypes were utilized in the multienvironmental trials. Based on multivariate analysis and GT biplot, Pod number in plant (PNP) and plant height (PH) were detected as the most essential selection criteria for grain yield enhancement in oilseed rape. The GT biplot facilitated visual comparisons of the traits, but was flawed in genotype ranking due to the negative correlation of some traits with grain yield. Genotype assessment based on grain yield composition with quantitative traits through GYT methodology eliminated this defect. Accordingly, eight above‐average genotypes were detected. Of them, G10, G5, G14, G11, and G2 were ranked above the RGS003 check cultivar, while G10 and G5 ranked above both check cultivars (Dalgan AND RGS003), as the first and second determined genotypes. Therefore, genotypes G10 and G5 were detected as the best genotypes with the highest grain yield and outstanding quantitative traits.

## FUNDING INFORMATION

This research received no specific grant from any funding agency in the public, commercial, or not‐for‐profit sectors.

## CONFLICT OF INTEREST STATEMENT

The authors declare that they have no conflict of interest.

## INFORMED CONSENT

Written informed consent was obtained from all study participants.

## Data Availability

The data that support the findings of this study are available from the corresponding author upon reasonable request.
